# Oral nystatin for the prevention of antibiotic-related fungal peritonitis in peritoneal dialysis patients: a systematic review and meta-analysis of randomized and observational studies

**DOI:** 10.1093/jacamr/dlag006

**Published:** 2026-02-03

**Authors:** Antonio Russo, Nicola Coppola, Carlo Torti, Enrico Maria Trecarichi

**Affiliations:** Dipartimento di Scienze Della Vita, Della Salute e Delle Professioni Sanitarie, Università Degli Studi Link, Rome, Italy; Dipartimento di Salute Mentale e Fisica e Medicina Preventiva, UOC Malattie Infettive, Università Degli Studi Della Campania ‘Luigi Vanvitelli’, Naples, Italy; Dipartimento di Scienze Mediche e Chirurgiche, UOC Malattie Infettive, Fondazione Policlinico Universitario Agostino Gemelli IRCCS, Rome, Italy; Dipartimento di Sicurezza e Bioetica, Università Cattolica del Sacro Cuore, Sez. Malattie Infettive, Rome, Italy; Dipartimento di Scienze Della Vita, Della Salute e Delle Professioni Sanitarie, Università Degli Studi Link, Rome, Italy

## Abstract

**Objectives:**

Fungal peritonitis is a severe complication in peritoneal dialysis (PD) patients, frequently occurring after antibiotic exposure. This systematic review and meta-analysis assessed whether oral nystatin administered concomitantly with antibiotics reduces the incidence of antibiotic-related fungal peritonitis (ARFP) compared with no prophylaxis.

**Methods:**

Following PRISMA guidelines, MEDLINE, PubMed and Embase were searched up to 1 October 2025 for randomized and observational studies enrolling adult PD patients who received systemic antibiotics with or without oral nystatin prophylaxis. The primary outcome was the odds of ARFP. ORs were pooled using random-effects models. This systematic review and meta-analysis was registered in PROSPERO (CRD420251154535).

**Results:**

Four prospective observational studies and one randomized controlled trial (RCT), comprising 2060 PD patients, were included. ARFP yielded 13 events among 1044 peritonitis episodes in the nystatin-treated patients versus 31 among 1016 in the control group (OR 0.53; 95% CI, 0.18–1.57). In the two studies (one observational and one RCT) reporting prescription-level data, nystatin was significantly associated with ARFP reduction (7 events out of 2205 prescriptions in the prophylaxis group versus 16/1724; OR 0.35; 95% CI, 0.15–0.87).

**Conclusions:**

Oral nystatin administered during antibiotic therapy was associated with a numerically lower rate of ARFP in PD patients, although all pooled estimates were based on unadjusted data and a limited number of events, thus precluding any certain causal inference. However, given its favourable safety profile and local action, nystatin may represent a valid candidate for a prophylactic strategy warranting further evaluation in contemporary, adequately powered randomized trials.

## Introduction

Fungal peritonitis represents one of the most serious complications in patients undergoing peritoneal dialysis (PD), often leading to catheter removal, transfer to haemodialysis and increased mortality.^1–3^ Exposure to antibiotics is a well-recognized risk factor, as it alters the intestinal and peritoneal microbiota and facilitates fungal overgrowth.^4–6^ For this reason, many centres have implemented antifungal prophylaxis during antibiotic therapy in PD patients. Overall, a Cochrane meta-analysis of the two randomized controlled studies of antifungal prophylaxis, one with nystatin and the other with fluconazole, showed an overall risk ratio of 0.28 (95% CI, 0.12–0.63) for fungal peritonitis occurring after a patient has had an antibiotic course (antibiotic-related fungal peritonitis, ARFP).^7^ Based on these results, current guidelines by the International Society for Peritoneal Dialysis (ISPD) recommend either fluconazole or nystatin as preferred agents to be co-prescribed whenever PD patients receive antibiotic therapy of any duration and regardless of the indication, in order to prevent fungal peritonitis.^8^ A recent meta-analysis confirmed the efficacy of fluconazole versus no prophylaxis in preventing the incidence of fungal peritonitis.^9^ Nystatin, a non-absorbable polyene antifungal, is among the most commonly used agents due to its low cost, minimal systemic toxicity and oral administration. It is not absorbed and acts locally in the gastrointestinal tract, reducing fungal colonization and potentially lowering the risk of translocation and subsequent peritonitis. Despite its widespread use and its possible advantages in terms of toxicity, drug–drug interactions and antifungal resistance, the evidence supporting nystatin prophylaxis is heterogeneous. One randomized trial and observational studies have reported varying results, with some demonstrating a protective effect whereas others show limited or no benefit, and no systematic review and/or meta-analysis on this important topic has ever been performed.^10–14^

By synthesizing available evidence, this study aimed to clarify whether prophylaxis with oral nystatin, administered concomitantly with antibiotic therapy, reduces the incidence of ARFP in PD patients compared with no prophylaxis.

## Methods

### Search strategy

We conducted a systematic review and meta-analysis of randomized controlled trials (RCTs) and observational studies comparing the efficacy of prophylaxis with nystatin to prevent fungal peritonitis in PD patients who underwent antibiotic treatment compared with PD patients who did not receive fungal prophylaxis during antibiotic treatment. The study protocol was developed in accordance with the PRISMA guidelines and minimal requirements for reporting of systematic reviews including observational studies (Table S1, available as Supplementary data at *JAC-AMR* Online), and registered in the International Prospective Register of Systematic Reviews (PROSPERO; registration number CRD420251154535).^15^ We screened original reports using MEDLINE, PubMed and Embase up to 1 October 2025, involving both medical subject heading (MeSH) terminology and relevant keywords to identify articles that evaluate the efficacy of nystatin prophylaxis during antibiotic treatment in preventing fungal peritonitis in PD patients. The following items were used to search the studies: ‘nystatin’ and ‘peritoneal dialysis’ or ‘CAPD’. In addition, the reference lists of all studies retrieved as full papers were manually searched to identify any other study that might be eligible for inclusion. All studies included had to fulfil the following criteria: (i) enrolling adult or paediatric patients undergoing PD; (ii) including patients who received systemic antibiotic therapy for any indication; (iii) including both patients who received oral nystatin prophylaxis administered during antibiotic treatment (any indication, dose, frequency or duration) and patients who did not receive any prophylaxis during antibiotic treatment; (iv) reporting at least one relevant endpoint, preferably incidence of fungal peritonitis, with sufficient data to calculate or extract effect measures [risk ratio (RR), OR, incidence rate ratio (IRR)]; (v) randomized controlled trials (RCTs), quasi-experimental studies, prospective or retrospective cohort studies, case-control studies; and (vi) to be published in English as a full paper. The exclusion criteria of the meta-analysis were: (i) studies without a comparator group; (ii) studies reporting only bacterial peritonitis without separate data for fungal peritonitis; (iii) reviews, editorials, expert opinions or conference abstracts without extractable data (unless additional information could be obtained from authors), case series, case report; (iv) duplicate publications of the same dataset (the most complete/updated version will be retained); (v) fewer than five patients in nystatin prophylaxis. Minimal requirements for performance and reporting of systematic reviews, including observational studies, based on a modified PRISMA template, are reported in Table S1. If required, the authors of studies not reporting clearly defined outcomes were contacted to retrieve the information.

### Study selection

Two researchers (A.R., E.M.T.) independently screened the title, abstract and keywords of all citations to identify potentially eligible articles. Reasons for the exclusion of any study were recorded independently. Thereafter, studies selected during the first screening were retrieved as full texts to be assessed for inclusion. In the case of disagreement, the reviewers re-evaluated the article together; if necessary, one of the other two authors (N.C.) was consulted.

### Data extraction

Two authors (A.R., E.M.T.) working independently extracted the data using a data-collection form previously established. The following relevant information was collected from every article, if available: the last name of the first author, year of publication, country where the study was conducted, study design, type of peritoneal dialysis, calendar period of enrolment, number of patients included, mean age and sex distribution, cause of antibiotic treatment, definition adopted for ARFP, number of patients who received nystatin prophylaxis, and timing of prophylaxis. Regarding outcomes, the total number of episodes of ARFP and the total number of peritonitis episodes and antibiotic prescriptions in treatment and control groups, were collected. The corresponding author was contacted to see if additional data were needed to identify patients enrolled in the study. If more than one study enrolled the same patient population, only the most complete article was included in the analysis.

### Quality assessment

Two reviewers (A.R. and E.M.T.) independently performed the quality appraisal of each study. Risk of bias assessment of RCTs was conducted using the Cochrane Risk of Bias Tool.^16^ The Newcastle-Ottawa Scale (NOS) was used to assess the quality of observational studies.^17^ The articles based on the NOS score were divided into three groups: 0–3 (fair), 4–6 (moderate), and 7–9 (good). In the case of discrepancies between the researchers the quality assessment was jointly re-evaluated. If necessary, one of the other two authors (C.T.) was consulted to reach the final decision.

### Predictors and outcomes

The primary outcome of this meta-analysis was the odds of ARFP estimated at the level of peritonitis episodes, which was prespecified as the primary analysis in order to ensure consistency across studies and maximize data availability. However, a secondary, exploratory analysis was planned a priori using antibiotic prescriptions as the unit of observation. This approach was considered conceptually relevant because antifungal prophylaxis with nystatin is prescribed in direct temporal relation to antibiotic exposure, and the biological rationale for ARFP prevention is linked to each antibiotic course rather than to peritonitis episodes occurring during follow-up. However, given that prescription-level data were available only in a subset of studies, and to avoid over-reliance on incomplete reporting, this analysis was predefined as secondary and hypothesis-generating.

### Quantitative data synthesis

ORs were used as the meta-analytic measure of association between therapy and the incidence of events. For each study, a proportion of patients with an event for the two therapeutic approaches was used to calculate ORs using a 2 × 2 table. For observational studies, we predefined the adjusted effect estimates to be extracted when available. Specifically, we planned to collect adjusted ORs, adjusted RRs, or adjusted HRs with their corresponding 95% CIs and the variables included in the adjustment model. Where available, adjusted ORs were converted to the log scale to be meta-analysed and then exponentiated for reporting. Heterogeneity between the studies was assessed using the Q statistic and *I*^2^. *I*^2^ values between 25% and 49% indicate low heterogeneity, between 50% and 75% indicate moderate heterogeneity, and an *I*^2^ value of 75% or above indicates high heterogeneity; a *P* value for the Q statistic <0.10 was considered significant.^18^ Considering the different population sizes of the studies, we chose a random-effect size; when *I*^2^ was <50% and *P* ≥ 0.1, a fixed-model was also fitted. Where not specified, tests were two-sided, and *P* values <0.05 were considered significant. Sensitivity analyses were planned to evaluate the robustness of findings by (i) separating RCTs from observational studies, (ii) stratifying studies by risk of bias, and (iii) examining differences according to the population and outcome definition (i.e. incidence of fungal peritonitis versus antibiotic prescription episodes). A funnel plot was used to visually assess potential publication bias, and Egger’s regression test was applied to statistically evaluate funnel plot asymmetry. All statistical analyses were performed using Stata/IC, version 16 software (Stata Corporation, College Station, TX, USA).^19^

## Results

**Figure 1.**
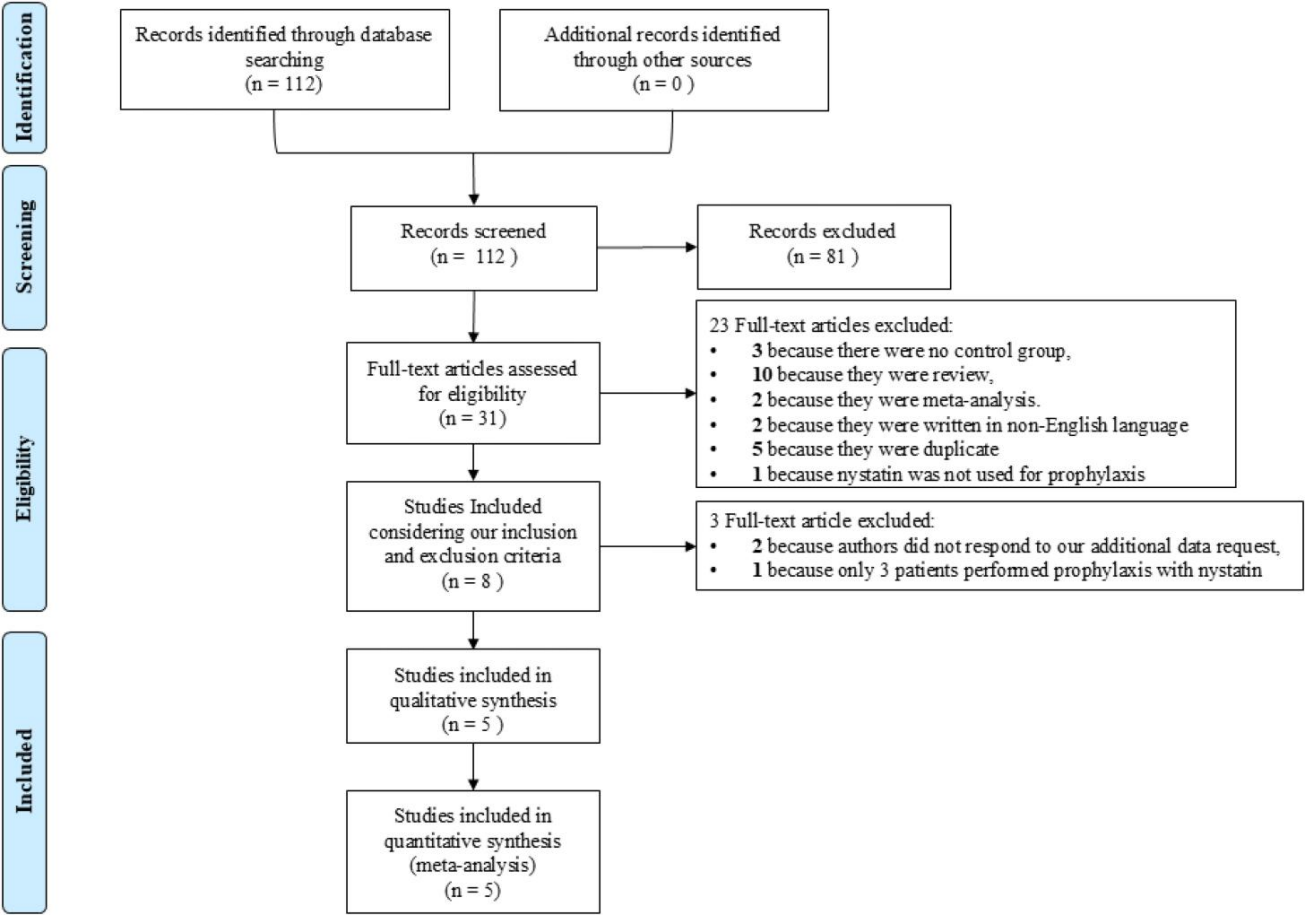
PRISMA flow chart of studies included.

Figure 1 shows the PRISMA flow diagram for article selection. A total of 112 citations from the electronic search were identified; among them, 81 were excluded on the basis of the title and abstract, and 23 for other causes. Of the eight full articles selected, five had all data needed for our meta-analysis; one was excluded due to a small sample size in the nystatin prophylaxis group (<5 patients); for the two remaining, further data were needed and these were requested from the corresponding authors. Unfortunately, these authors replied that they were unavailable to provide the requested data.

Therefore, a total of five studies were included in the analysis and are described in detail in Table 1.^10–14^ One was an RCT,^10^ the others were observational prospective studies.^11–14^ Overall, the studies were conducted between 1979 and 2005, reflecting PD practices and antibiotic prescribing patterns that differ substantially from contemporary clinical settings, in China (one in Hong Kong), Switzerland, the USA and the UK. All studies were conducted in PD populations. The number of patients enrolled ranged from 108 to 801. The mean age varied from 48.4 to 65.9 years, and sex distribution was reported in two studies.^10,13^ Causes of antibiotic treatment included peritonitis, exit-site infection or other indications. The definition of ARFP differed across studies, ranging from 1 month to 3 months after antibiotic exposure depending on the protocol; in two studies, this information was not reported. The number of patients receiving nystatin prophylaxis varied between 93 and 481. Prophylaxis was started concomitantly with antibiotic therapy, and in three studies continued for more days after the last dose of aminoglycosides or vancomycin.^10,13,14^ The number of events included in the analysis is shown in Table 2. In particular, the total number of peritonitis episodes varied between 94 and 499 in controls, and between 99 and 499 in prophylaxis groups. The total number of antibiotic prescriptions was reported in only two studies and ranged from 420 to 1753 in the prophylaxis groups and from 187 to 1304 in the control groups. ARFP episodes ranged from 0 to 4 in the prophylaxis group and from 2 to 10 in control groups. Quality assessments are reported in Tables S2 and S3. Observational studies included showed a low risk of bias, whereas the RCT showed some concerns (Table S3).

**Table 1.** Characteristics of studies included in the meta-analysis

Author	Year	Country	Design	Type of peritoneal dialysis	Study period	Total number of patients	Mean age (SD)	Male sex, *n* (%)	Indication/s for antibiotic treatment	Definition of antibiotic-related fungal peritonitis	Number of patients in nystatin prophylaxis group	Number of total antibiotic prescription episodes	Timing of nystatin prophylaxis
Wong *et al.*^13^	2007	China	Prospective	CAPD	1995–2005	801	60.4 (11.4) in CG; 61 (12.1) in TG	172 (53.7) in CG; 246 (51.1) TG	Any	Exposure to antibiotics within 1 mo of episode of FP	481 (13 725 patient-months)	3057	Throughout the whole course of antibiotic plus additional 7 d if vancomycin was used
Zàruba *et al.*^14^	1991	Switzerland	Prospective	CAPD	1979–1989	108	50.9	NR	Any	NR	93 (2102 patient-months)	NR	Throughout the whole course of antibiotic
Thodis *et al.*^11^	1998	USA	Prospective	CAPD	1996–1997	240	65.9 (8) in CG; 65.9 (8) in TG	NR	Any	Exposure to antibiotics within 60 d of episode of FP	240 (2400 patient-months)	NR	Throughout the whole course of antibiotic plus additional 5 d
Lo *et al.*^10^	1996	Hong Kong (China)	Randomized controlled trial	CAPD	1991–1993	397	48.5 (14.2) in CG; 48.4 (14.5) in TG	98 (49.5) in CG. 86 (43.2) in TG	NR	Exposure to antibiotics within 3 mo of episode of FP	199	872	Throughout the whole course of antibiotic, plus additional 3 d after the last dose of aminoglycosides or plus additional 7 d after last dose of vancomycin
Williams *et al.*^12^	2000	UK	Prospective	NR	1997–1999	3911 patient-months (TG); 2124 patient-months (CG)	NR	NR	Peritonitis and exit-site infection	NR	NR	NR	Throughout the whole course of antibiotic

CAPD, continuous ambulatory peritoneal dialysis; CG, no nystatin prophylaxis patients; FP, fungal peritonitis; NR, not reported; TG, nystatin prophylaxis group.

**Table 2.** Variables extrapolated from the studies included in the meta-analysis

Author	Year	Total number of AB prescriptions CG/TG	Number of peritonitis in CG/TG	Number of FP in CG/TG	Number of patients with FP considered antibiotic-related in CG	Number of patients with antibiotic-related FP who received prophylaxis with nystatin in TG
Wong *et al.*^13^	2007	1304/1753	414/499	14/13	10	4
Zàruba *et al.*^14^	1991	NR	94/127	10/4	10	0
Thodis *et al.*^11^	1998	NR	133/99	6/12	3	2
Lo *et al.*^10^	1996	420/452	188/216	12/4	6	3
Williams *et al.*^12^	2000	NR	187/103	2/3	2	2

AB, antibiotics; CG, control group; FP, fungal peritonitis; NR, not reported; TG, nystatin group (treatment group).

**Figure 2.**
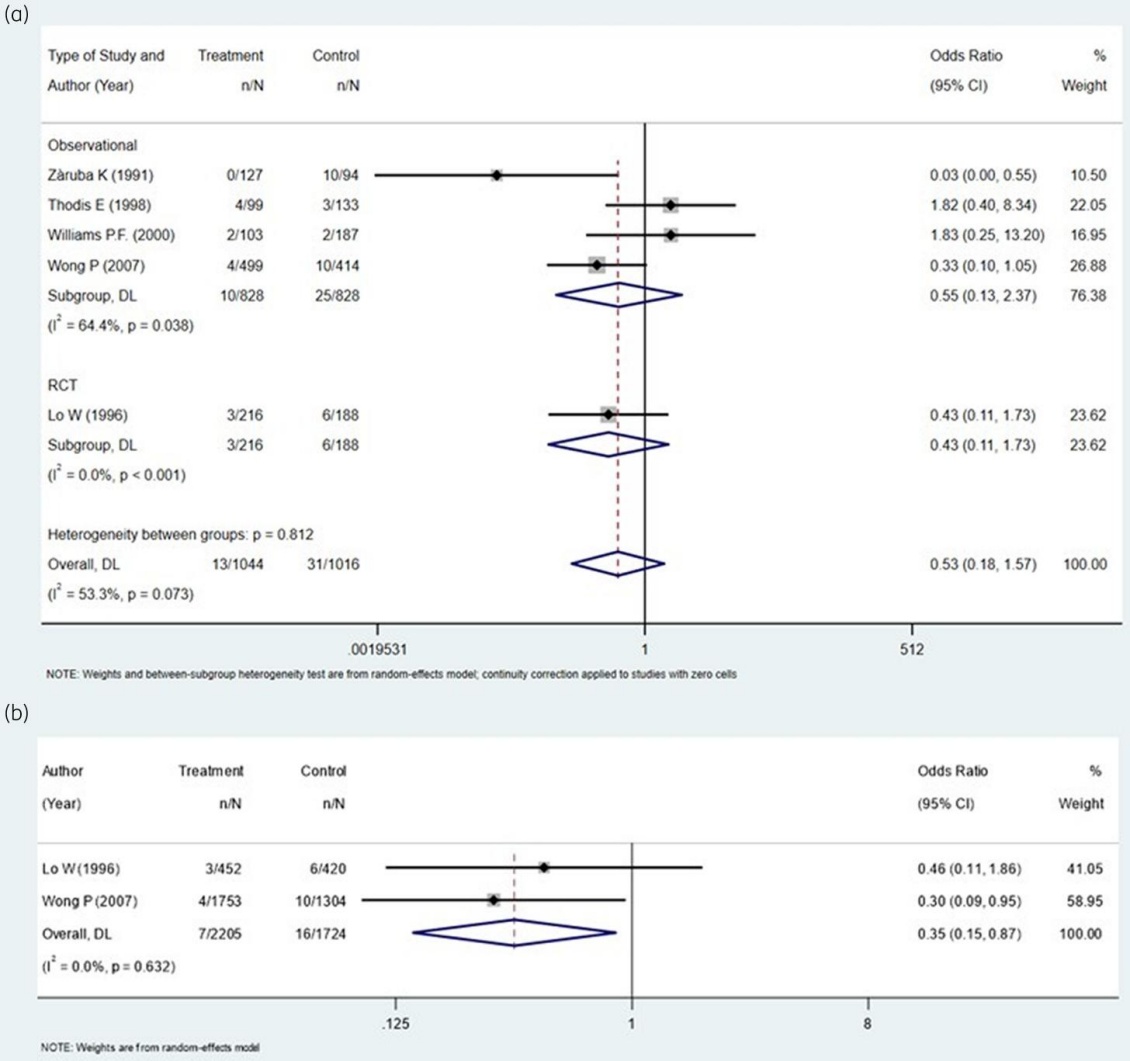
Forest plots of ORs. (a) Forest plot of ORs of antibiotic-related fungal peritonitis (ARFP) in patients receiving or not receiving nystatin prophylaxis considering all peritonitis episodes during follow-up. (b) Forest plot of ORs of ARFP in patients receiving or not receiving nystatin prophylaxis considering the total number of antibiotic prescriptions. Vertical dashed lines correspond to the summary odds ratio estimates, and the width of each blue rhomboid reflects the associated 95% confidence interval.

The outcomes of the meta-analysis are shown in Figure 2 and Tables S3 and S4. All five included studies reported the number of ARFP events, which were 13 in the prophylaxis group and 31 in the control group. Considering the total number of peritonitis episodes in each population, grouped by the design of the study, the OR of ARFP in observational studies (828 patients in the nystatin group and 828 in the control group) was 0.55 (95% CI, 0.13–2.37; *P* = 0.038; *I*^2^=64.4%), whereas the OR of ARFP in the RCT (216 patients in the nystatin group and 188 in the control group) was 0.43 (95% CI, 0.11–1.73; *P* < 0.001; *I*^2^=0.0%) (Figure 2, Table S4). Considering both observational studies and the RCT, the OR of ARFP was 0.53 (95% CI, 0.18–1.57; *P* = 0.073; *I*^2^ = 53.3%) (Figure 2, Table S4).

**Figure 3.**
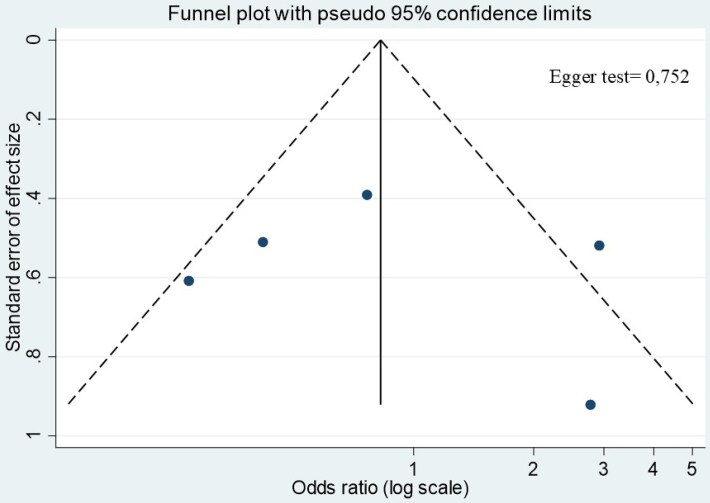
Funnel plot of included studies (exploratory assessment; limited interpretability due to small number of studies).

As prespecified in the Methods, analyses based on antibiotic prescriptions were conducted as secondary and exploratory analyses. Only in two studies (one observational,^13^ one RCT^10^) were ARFP episodes analysed with antibiotic prescriptions as units of analysis, and were included for this purpose with a total of 7 events out of 2205 prescriptions in the prophylaxis group and 16 out of 1724 in the control group (Figure 2). The random-effects model showed an OR of 0.35 (95% CI, 0.15–0.87; *P* = 0.632; *I*^2^ = 0%, Figure 2b, Table S5), which was confirmed in the fixed-effect model (OR 0.35; 95% CI, 0.15–0.86; *P* = 0.632; *I*^2^ = 0%; Table S5). Since none of the included observational studies reported adjusted effect estimates, all pooled analyses were necessarily based on crude ORs derived from 2 × 2 tables, which are inherently susceptible to confounding by indication and baseline imbalances. There was substantial heterogeneity across studies in the definition of ARFP, with time windows ranging from 1 to 3 months after antibiotic exposure, and in the analytical unit adopted (peritonitis episodes versus antibiotic prescriptions), which may have contributed to between-study heterogeneity and limited comparability of pooled estimates. As such, the pooled association reflects unadjusted data and may be influenced by confounding. When considering all the studies included in the analysis, funnel plot inspection did not reveal clear asymmetry; however, given the very small number of included studies, this assessment should be regarded as exploratory and of limited interpretive value (Figure 3).

## Discussion

The present systematic review and meta-analysis focused on the efficacy of oral nystatin prophylaxis administered concomitantly with antibiotic therapy in PD patients in order to prevent ARFP. Across four prospective studies and one RCT, the pooled results demonstrated a non-significant trend towards reduced incidence of ARFP in the nystatin group compared with controls, considering the total amount of peritonitis episodes in PD patients. However, when ARFP episodes were analysed using the total of antibiotic prescriptions (and not total of peritonitis events), as reported in only two of the included studies, the effect of nystatin was significantly associated with a lower frequency of ARFP in crude analyses, with an OR of 0.35 (95% CI, 0.15–0.87), indicating nearly a two-thirds reduction in risk. Since the actual effectiveness of an antimicrobial prophylaxis in preventing a specific type of infection should be evaluated as the number of events (infections) out of the total number of antimicrobial prophylaxis prescriptions, the latter analysis may conceptually better reflect the clinical context in which antifungal prophylaxis is prescribed; however, given that this finding is derived exclusively from crude, unadjusted data and a very small number of events, results should still be interpreted with caution. The efficacy of fluconazole prophylaxis has been more extensively studied, and a recent meta-analysis including six studies (one RCT and five observational cohorts) representing a total of 4515 peritonitis episodes [1098 (24.8%) in the fluconazole prophylaxis group and 3417 (75.6%) in the no prophylaxis group] confirmed that fluconazole prophylaxis significantly reduced the risk of fungal peritonitis compared with no prophylaxis (OR 0.22; 95% CI, 0.12–0.41), with consistent findings across subgroups and sensitivity analyses.^9^ These results establish fluconazole as an effective option for antifungal prophylaxis in PD patients. However, important issues regarding the use of fluconazole with this indication can be raised. Firstly, fluconazole, as a systemic triazole antifungal, undergoes hepatic metabolism via the cytochrome P450 system and is a potent inhibitor of CYP2C9, CYP2C19 and CYP3A4. Consequently, it is prone to significant pharmacokinetic drug–drug interactions with a wide spectrum of commonly prescribed medications, including anticoagulants, antiepileptics, calcineurin inhibitors and certain statins.^9^ Such interactions can complicate management in PD patients, who are frequently elderly, polymedicated and burdened with multiple comorbidities.^20^ Secondly, but no less importantly, toxicity and selection of azole-resistant fungal strains leading to subsequent severe infections can occur.^21,22^ In contrast, because it is not systemically absorbed, nystatin is virtually free from systemic toxicity and pharmacokinetic drug–drug interactions, and acts locally within the gastrointestinal tract.^10^ These characteristics could represent a considerable advantage in the setting of PD population. Although the protective efficacy of nystatin in preventing fungal peritonitis has shown variability across individual studies, with some reporting significant benefit and others no difference, our pooled analysis suggests a possible association between nystatin prophylaxis and lower rate of ARFP in PD patients versus controls without prophylaxis, similar to fluconazole, as reported in the aforementioned meta-analysis.^9^ Therefore, our findings support the current ISPD guidelines in recommending either fluconazole or nystatin as preferred agents to be co-prescribed in order to prevent ARFP whenever PD patients receive antibiotic therapy courses.^8^

In line with recent methodological recommendations for systematic reviews including observational studies, we defined a priori the characteristics of an ideal observational study capable of addressing the research question with minimal risk of confounding. Such a study would: (i) clearly define exposure and comparator groups within the same clinical population and time window; (ii) adopt a standardized definition of ARFP; (iii) ensure correct temporal alignment between exposure and outcome; and (iv) report adjusted effect estimates controlling for a minimal set of predefined confounders. Unfortunately, none of the observational studies included in the present study fulfilled these methodological criteria, particularly with regard to adjustment for confounding. Therefore, the available evidence could reflect only unadjusted associations, which may be subject to substantial bias. Because all studies, four observational and one RCT, included in our analyses report only crude estimates, and because confounding by indication is a major source of bias in this clinical context, the pooled association should not be interpreted as evidence of a causal effect. The direction and magnitude of the association may be influenced by patient characteristics that were unevenly distributed between groups and were not controlled for. Accordingly, when applying the GRADE framework, the certainty of evidence was rated as low to very low, primarily due to serious imprecision related to the small number of events and wide CIs, as well as risk of confounding in observational studies. Therefore, the observed associations should not be interpreted as proof of a causal protective effect.

This study has several important limitations that should be acknowledged when interpreting the results. Firstly, none of the included observational studies reported adjusted effect estimates. Consequently, all pooled analyses are based on crude ORs, which are highly susceptible to confounding by indication and other baseline imbalances. Factors such as antibiotic type and duration, severity of infection, prior peritonitis history, comorbidity burden and centre-specific prophylaxis policies may have influenced both the likelihood of receiving nystatin prophylaxis and the risk of developing ARFP. As these factors were not controlled for, the observed associations cannot be interpreted as causal. Secondly, definitions of ARFP varied substantially, with observation windows ranging from 1 to 3 months after antibiotic exposure, and studies differed in whether outcomes were analysed per peritonitis episodes or per antibiotic prescriptions. Although the primary meta-analysis based on peritonitis episodes showed a non-significant association, statistical significance emerged only in a secondary analysis restricted to two studies reporting prescription-level data. Given the limited number of studies and events contributing to this secondary analysis, this finding should be regarded as exploratory and hypothesis-generating rather than confirmatory. Thirdly, the total number of outcome events was very limited. Across all included studies, only 44 episodes of ARFP were observed, with 13 events in the nystatin group and 31 in the control group. Such sparse data substantially reduce statistical power and increase the risk of imprecise and unstable effect estimates, as reflected by the wide CIs in several pooled analyses. Consequently, the absence of statistical significance in some analyses may reflect limited power rather than lack of a true effect. Fourthly, all the included studies are dated, having been conducted between 1979 and 2007, a period preceding substantial changes in PD practice. Since then, major advances have occurred in catheter technology, connection systems, exit-site care, antibiotic stewardship and antifungal resistance patterns, as well as shifts in the epidemiology of fungal peritonitis. These changes may have modified both the baseline risk of ARFP and the relative effectiveness of antifungal prophylaxis strategies, thereby limiting the applicability of the present findings to current clinical practice. Finally, the small number of included studies and outcome events limited our ability to perform formal subgroup or moderator analyses to investigate heterogeneity, beyond the predefined stratified analyses by study design and unit of analysis. However, to date no studies have investigated whether nystatin could be preferable to fluconazole for this purpose, based on the above reported possible advantages. Future research should address this gap by conducting adequately powered RCTs directly comparing nystatin versus fluconazole in PD patients, stratified by antibiotic type and comorbidity burden. Such trials could be essential to define a personalized approach, balancing efficacy with safety and tolerability.

Both fluconazole and nystatin are recommended as options for antifungal prophylaxis in PD patients receiving systemic antibiotics, in line with current ISPD guidelines. In our review, the available observational studies and RCT suggested an association between nystatin prophylaxis and a lower occurrence of ARFP; however, the limited number of events and the resulting imprecision prevent definitive conclusions. Moreover, these estimates could be derived exclusively from crude (unadjusted) data and are therefore susceptible to confounding by indication and other biases inherent to observational designs. As such, the observed association should not be interpreted as evidence of a causal protective effect. In conclusion, the available observational studies and the single randomized trial suggest a possible association between nystatin prophylaxis and a lower occurrence of ARFP. However, given the low to very low certainty of evidence and the substantial methodological limitations, these findings should be considered hypothesis-generating and do not provide confirmatory evidence of clinical effectiveness. To determine whether nystatin may offer clinical advantages over fluconazole, particularly regarding drug–drug interactions, tolerability and the potential to limit azole resistance, adequately powered RCTs directly comparing the two agents are warranted. Future high-quality studies are essential to clarify whether the observed association reflects a true preventive effect and to guide personalized prophylaxis strategies in PD patients.

## Supplementary Material

dlag006_Supplementary_Data

## Supplementary data

Tables S1 to S5 are available as Supplementary data at *JAC-AMR* Online.
