# Supplementary material for: Oral nystatin for the prevention of antibiotic-related fungal peritonitis in peritoneal dialysis patients: a systematic review and meta-analysis of randomized and observational studies
Source: JAC Antimicrob Resist. 2026 Feb 3;8(1):dlag006. doi: 10.1093/jacamr/dlag006 (PMC12866647; doi:10.1093/jacamr/dlag006)
Supplement: dlag006_Supplementary_Data [file dlag006_supplementary_data.docx]

**Table S1**. Minimal requirements for reporting of systematic reviews including observational studies (extracted from recommendations included in the study Paul M et al. 2025 [Paul M, Olchowski J, Leibovici L. Systematic reviews of observational studies frequently conclude based on meta-analyses of biased results: standards must be improved. *J Clin Epidemiol* 2025; **184**:111840].

| **Item** | **Requirements** | **Page** |
| --- | --- | --- |
| Title | Denote study designs included in the systematic review | Page 1 |
| **Abstract** |  |  |
| Eligibility criteria | Define study design for inclusion | Page 2 |
| Included studies | Report on the study designs on which results are based | Page 2 |
| Interpretation/conclusions | Wording denoting effects should not be used to conclude on data from observational studies | Page 2 |
| **Background** | Provide the rationale of including observational studies in the systematic review Preferably describe the causal model linking the exposure to the outcome in the observational studies (eg, directed acyclic graph) | Page 3 |
| **Methods** |  |  |
| Inclusion criteria | Define the inclusion of observational studies and denote the study designs eligible, including minimal requirements from the observational studies, as relevant | Page 4, paragraph 2.1 |
| Data items | Define the data that will be extracted from the observational studies. This should address the extraction of adjusted results, including the acceptable methods or minimal requirements from the adjusted analyses | Page 5, paragraph 2.3 |
| Risk of bias assessment | Define and report the important confounders for the topic of the systematic review based on the causal model and use the predefined confounders when assessing risk of bias | Page 5, paragraph 2.4 |
| Effect measures | Define the adjusted effect measures that will be extracted from observational studies | Page 6, paragraph 2.6 |
| Data synthesis | Methods of compiling adjusted association estimates should be defined, addressing the use of adjusted and unadjusted results and the handling of studies that do not perform an adjusted analysis. Describe sensitivity analyses to examine assumptions and imputations Describe whether and how different effect measures were combined (eg, odds ratios, hazard ratios) | Page 7, paragraph 2.6 |
| **Results** |  |  |
| Included studies | Report whether each study addressed the biases defined per protocol. Studies can be grouped by:  a. Studies in which an analysis that takes into account the important biases.  b. Studies in which an adjusted analysis was done, but the important factors were not accounted for  c. Studies for which only the raw data exist | Page 8, paragraph 3 |
| Risk of bias reporting | Use the protocol-defined confounders for assessment of bias due to confounding (ROBINS-I V2) or comparability (NOS) or any other risk score. The risk of bias of the association estimates (‘‘effects’’) used in the meta-analysis should be reported | Page 9, paragraph 3 |
| Results synthesis | Define whether analyses are based on adjusted or unadjusted results Separate meta-analyses of adjusted and unadjusted results | Not applicable |
| Certainty of the evidence | Consider whether grading applies to adjusted or unadjusted results; and report the GRADE relevant to the data used in the meta-analyses | Page 10, paragraph 4 |

**Table S2**: Newcastle - Ottawa quality assessment scale cohort studies

|  |  | First author, year, [Reference n.] | Wong et al. 2007 ^13^ | Zàruba et al 1991 ^11^ | Thodis et al 1998 ^14^ | Williams P.F. et al ^12^ |
| --- | --- | --- | --- | --- | --- | --- |
| Domain 1: Selection | Representativeness of the exposed cohort | truly representative of the average PD patients in the community | X | X | X | X |
|  |  | somewhat representative of the average PD patients in the community |  |  |  |  |
|  |  | selected group of users e.g. nurses, volunteers |  |  |  |  |
|  |  | no description of the derivation of the cohort |  |  |  |  |
|  | Selection of the non-exposed cohort | drawn from the same community as the exposed cohort | X | X | X | X |
|  |  | drawn from a different source |  |  |  |  |
|  |  | no description of the derivation of the non- exposed cohort |  |  |  |  |
|  | Ascertainment of exposure | secure record (eg surgical records) | X | X | X | X |
|  |  | structured interview |  |  |  |  |
|  |  | written self report |  |  |  |  |
|  |  | no description |  |  |  |  |
|  | Demonstration that outcome of interest was not present at start of study | yes | X | X | X | X |
|  |  | no |  |  |  |  |
| Comparability | Comparability of cohorts on the basis of the design or analysis | study controls for FP | X | X | X | X |
| Outcome | Assessment of outcome | independent blind assessment |  |  |  |  |
|  |  | record linkage | X | X | X | X |
|  |  | self report |  |  |  |  |
|  |  | no description |  |  |  |  |
|  | Was the follow-up long enough for outcomes to occur? | Yes | X | X | X | X |
|  |  | no |  |  |  |  |
|  | Adequacy of follow-up of cohorts | complete follow-up - all subjects accounted for |  |  |  |  |
|  |  | subjects lost to follow- up unlikely to introduce bias - small number lost |  |  |  |  |
|  |  | adequate (70%) follow up, or description provided of those lost) |  |  |  |  |
|  |  | follow up rate < 70% and no description of those lost |  |  |  |  |
|  |  | no statement | X | X | X | X |

**Table S3:** Revised Cochrane risk-of-bias tool for randomized trial included in the study.^10^

| **Domain** | **Signalling question** | **Response** |
| --- | --- | --- |
| **Bias arising from the randomization process** | 1.1 Was the allocation sequence random? | Y |
|  | 1.2 Was the allocation sequence concealed until participants were enrolled and assigned to interventions? | NI |
|  | 1.3 Did baseline differences between intervention groups suggest a problem with the randomization process? | PN |
|  | Risk of bias judgement | Some concerns |
| **Bias due to deviations from intended interventions** | 2.1.Were participants aware of their assigned intervention during the trial? | Y |
|  | 2.2.Were carers and people delivering the interventions aware of participants' assigned intervention during the trial? | Y |
|  | 2.3. If Y/PY/NI to 2.1 or 2.2: Were there deviations from the intended intervention that arose because of the experimental context? | PN |
|  | 2.4 If Y/PY to 2.3: Were these deviations likely to have affected the outcome? | NA |
|  | 2.5. If Y/PY/NI to 2.4: Were these deviations from intended intervention balanced between groups? | NA |
|  | 2.6 Was an appropriate analysis used to estimate the effect of assignment to intervention? | PY |
|  | 2.7 If N/PN/NI to 2.6: Was there potential for a substantial impact (on the result) of the failure to analyse participants in the group to which they were randomized? | NA |
|  | Risk of bias judgement | Low |
| **Bias due to missing outcome data** | 3.1 Were data for this outcome available for all, or nearly all, participants randomized? | Y |
|  | 3.2 If N/PN/NI to 3.1: Is there evidence that result was not biased by missing outcome data? | NA |
|  | 3.3 If N/PN to 3.2: Could missingness in the outcome depend on its true value? | NA |
|  | 3.4 If Y/PY/NI to 3.3: Is it likely that missingness in the outcome depended on its true value? | NA |
|  | Risk of bias judgement | Low |
| **Bias in measurement of the outcome** | 4.1 Was the method of measuring the outcome inappropriate? | PN |
|  | 4.2 Could measurement or ascertainment of the outcome have differed between intervention groups? | N |
|  | 4.3 Were outcome assessors aware of the intervention received by study participants? | PY |
|  | 4.4 If Y/PY/NI to 4.3: Could assessment of the outcome have been influenced by knowledge of intervention received? | PN |
|  | 4.5 If Y/PY/NI to 4.4: Is it likely that assessment of the outcome was influenced by knowledge of intervention received? | NA |
|  | Risk of bias judgement | Low |
| **Bias in selection of the reported result** | 5.1 Were the data that produced this result analysed in accordance with a pre-specified analysis plan that was finalized before unblinded outcome data were available for analysis? | PN |
|  | 5.2 ... multiple eligible outcome measurements (e.g. scales, definitions, time points) within the outcome domain? | PN |
|  | 5.3 ... multiple eligible analyses of the data? | PN |
|  | Risk of bias judgement | Some concerns |
| **Overall bias** | Risk of bias judgement | Some concerns |

**Table S4**: Summary of meta-analysis results in the efficacy of nystatin prophylaxis considering all diagnosis of peritonitis.

| Outcome | No. of patients treatment/control group | No. (%) of events treatment/control group | OR (efficacy) | 95% Confidence Interval (efficacy) | Heterogeneity test (I^2^, %; p) |
| --- | --- | --- | --- | --- | --- |
| Fungal peritonitis defined AR including only observational study | 828/828 | 10/25 | 0.55 | 0.13-2.37 | 64.4, 0.038 |
| Fungal peritonitis defined AR including only Randomized controlled trial | 216/188 | 3/6 | 0.43 | 0.11-1.73 | 0.00, <0.001 |
| Fungal peritonitis defined AR including both observational study and randomized controlled trial | 1044/1016 | 13/31 | 0.53 | 0.18-1.57 | 53.3, 0.073 |

Footnotes: No., numbers; (%), percentage; OR, odds ratio; AR, antibiotic-related.

**Table S5**: Summary of meta-analysis results in the efficacy of nystatin prophylaxis considering all antibiotic prescriptions, including observational studies and randomized controlled trial.

| Outcome | No. of patients treatment/control group | No. (%) of events treatment/control group | OR (efficacy) | 95% Confidence Interval (efficacy) | Heterogeneity test (I^2^, %; p) |
| --- | --- | --- | --- | --- | --- |
| Fungal peritonitis defined AR (random model) | 2205/1724 | 7/16 | 0.35 | 0.15-0.87 | 0.0, 0.632 |
| Fungal peritonitis defined AR (fixed model) | 2205/1724 | 7/16 | 0.35 | 0.15-0.86 | 0.0, 0.632 |

Footnotes: No., numbers; (%), percentage; OR, odds ratio; AR, antibiotic-related.
